# Erratum

**DOI:** 10.1177/21501327211057676

**Published:** 2022-03-21

Erratum to: Hack B, Timalsina U, Tefera E, et al. Oral Prescription Opioids as a High-Risk Indicator for Hepatitis C Infection: Another Step Toward HCV Elimination. Journal of Primary Care & Community Health. January 2021. doi:10.1177/21501327211034379

Incorrect versions of Figures 1 and 2 for this article were uploaded at the time of publication. The correct Figures are now published.

